# Mental Health Providers’ Attitudes, Norms, and Beliefs About Cultural Humility in Service Delivery

**DOI:** 10.1007/s11414-025-09953-3

**Published:** 2025-06-18

**Authors:** Alexandria G. Bauer, Amudha Balaraman, Ayanna Gilmore

**Affiliations:** 1 Rutgers University-New Brunswick, 607 Allison Road, Piscataway, NJ 08854 USA; 2 The New School for Social Research, 6 E 16 St, New York, NY 10003 USA

**Keywords:** Multicultural orientation framework, Cultural humility, Mental health, Theory of planned behavior

## Abstract

Cultural humility is an important provider-level strategy for improving engagement and retention in mental health care. Yet, little research has used theoretical frameworks to examine beliefs and intentions to practice with cultural humility among mental health providers. The current study aimed to fill this gap by using the Theory of Planned Behavior (TPB) to qualitatively explore beliefs, norms, and attitudes regarding cultural humility practice among a sample of 16 professional mental health providers. Participants were predominately women (89.5%), with an average age of 38 years old (*SD* = 7.98). Participants completed a 30- to 60-min online interview. Thematic analysis was used to examine patterns and identify overarching themes within TPB constructs of behavioral, normative, and control beliefs. Themes within behavioral beliefs reflected improved practice, particularly for working with clients from marginalized groups. However, there was uncertainty in navigating cultural humility practice and concerns about potentially altering the client relationship. Referents for normative beliefs were clinical agencies, professional organizations, university training programs, and supervisors and mentors. Within control beliefs, themes for facilitators and barriers centered around lack of support from mental health systems, personal limitations and strengths, and professional development. This study furthers understanding of mental health providers’ attitudes and intentions to practice with cultural humility, as well as underscoring the nuances of when and how these practices are used. This research can help to address barriers and promote cultural humility practice among mental health providers across multiple fields.

**Supplementary Information:**

The online version contains supplementary material available at 10.1007/s11414-025-09953-3.

## Introduction

Across the USA, there are treatment gaps for people with mental health concerns. More than 50% of people with mental health disorders do not receive treatment, due to lack of access, insurance coverage, and availability of providers.^1^ For people from marginalized communities (e.g., based on race, ethnicity, sexual orientation, and/or gender identity), additional compounding barriers to treatment engagement and retention include mistrust of mental health systems, cultural presentation of symptoms, provider bias, and paucity of culturally relevant interventions.^1,2^ Mental health challenges are even greater among individuals who do not have their other needs met, including basic necessities for food, housing, finances, employment, and general health care.^3^ Cultural humility is one provider-level solution to closing treatment gaps by improving engagement and retention.

Cultural humility refers to a willingness and interest to learn about another’s culture, along with self-reflection of one’s own cultural identities, values, and beliefs. This consideration encompasses innumerable aspects of identity, from race, ethnicity, gender, and sexual orientation to physical appearance, languages spoken, locality or geographic region, spiritual or religious beliefs, health conditions, disability status, personal and professional roles (e.g., parent, student), and beyond. The process of self-exploration also incorporates a willingness to challenge biases and examine one’s relative social power, privilege, and hierarchical roles (e.g., client-provider, supervisor-supervisee).^4^ Cultural humility contrasts with similar concepts, like cultural competence, which were well-intended but have given rise to concerns about stereotyping and “othering” clients ^5^ or failing to acknowledge and shift power differentials.^4,6^Cultural humility stands apart, in that it is a lifelong process, rather than an endpoint or specific goal. It is one of three constructs in the multicultural orientation framework (MCO).^7^ The second element, cultural comfort, refers to one’s relative ease with having cultural conversations. The third element is cultural opportunities (i.e., taking advantage of chances to have conversations about cultural identity with others). Decades of research have described the development of the MCO and cultural humility, as well as its applications for mental health providers.^8-11^ Evidence suggests that practicing with cultural humility leads to improved treatment outcomes, including treatment retention and functional improvement.^8,12^

Despite efforts to promote these approaches, there are continued gaps in cultural humility practice. For example, people with mental health challenges continue to experience stigma and microaggressions from healthcare providers,^13,14^ and clients have reported racism and/or microaggressions from mental health providers on the basis of their race or gender identity.^15,16^ In particular, therapy clients from marginalized racial and ethnic groups (i.e., Black, Asian, Latina/o, and multiracial) experienced microaggressions more often than white clients.^17^ Racial microaggressions were subsequently associated with lower trust, weakened therapeutic alliance, and reduced treatment engagement.^18-20^ Furthermore, in a qualitative study of clients from marginalized racial/ethnic groups, participants who were unsatisfied with their mental health treatment reported that their providers displayed a lack of cultural humility (e.g., did not acknowledge or address the role of social privilege in clients).^21^ A study by Owen and colleagues found that lack of cultural humility among mental health providers was particularly damaging when combined with missed cultural opportunities, with these contexts leading to worse therapy outcomes.^12^ Notably, this study also found that client-rated cultural humility could be protective against missed opportunities, underscoring the need to more consistently implement cultural humility approaches in practice. Understanding providers’ intentions to engage in cultural humility practice may provide clearer pathways to promote these concepts within mental health treatment systems and thereby improve service delivery across populations, communities, and identities.

The Theory of Planned Behavior (TPB) provides a framework for understanding multiple influences on behavioral intentions and subsequent outcomes,^22^ such as behavioral engagement in cultural humility practices. Behavioral, normative, and control beliefs collectively predict intentions to engage in a behavior, and intentions are posited to predict actual behavioral engagement. Behavioral beliefs refer to one’s attitudes toward the behavior (e.g., whether it is good or bad, and relative level of value in it). Normative beliefs refer to perceived opinions of peers and others in influential roles (e.g., supervisors) and willingness to comply with their support or discouragement toward the behavior. Finally, control beliefs are one’s ability to engage in the behavior, made easier or more difficult by facilitators and barriers. Yet, little research has used the TPB to examine beliefs and intentions to practice with cultural humility among mental health providers.

The current study qualitatively explored beliefs, norms, and attitudes regarding cultural humility practice among a sample of professional mental health providers. Understanding TPB constructs, in the context of intentions to engage in cultural humility practices, can help to address barriers and promote cultural humility practice among mental health providers across multiple fields. This is paramount for promoting greater engagement and retention in mental health treatment, particularly for people from marginalized communities who have been mistreated by mental health systems.

## Methods

### Participants and Procedures

To be eligible for this qualitative pilot study (clinical trial number: not applicable), participants had to be adults (aged 18 and older); self-identify as a professional mental health service provider, such as psychologist, psychiatrist, therapist, or counselor; and currently provide mental health services at least part time. Clinicians in training (e.g., advanced doctoral students) were included in the sample. A convenience sample was recruited via announcements and flyers, which were posted online and emailed to national listservs of professional organizations. Eligible participants were emailed a link to provide written informed consent. All participants provided written informed consent. Participants were then emailed a link to complete an anonymous online survey and to schedule an interview.

Interviews were conducted by the first author via Zoom and lasted 30–60 min. Prior to the beginning the interview, the interviewer reviewed the purpose and goals of the study, and each participant chose a pseudonym (presented for analysis). Participants received a $20 virtual gift card after completing the interview. This study was reviewed and approved by the Rutgers University Institutional Review Board.

Sixteen mental health providers completed both the survey and interview. Three additional participants completed the survey but did not participate in an interview, either because they did not respond or declined to participate. Participants (described in Table 1) were primarily women and heterosexual, with an average age of 38 years (*SD* = 7.98, range = 28–59).

**Table 1:** Personal characteristics of mental health providers participating in the study

	*n*	%
Gender		
Men	1	5.3
Women	17	89.5
Non-binary/gender non-conforming	1	5.3
Race		
American Indian/Alaska Native	1	5.3
Asian	2	10.5
Black/African American	8	42.1
White	10	52.6
Other	1	5.3
Sexual orientation		
Heterosexual or straight	12	63.2
Bisexual	3	15.8
Asexual	3	15.8
Degree(s) obtained		
Doctor of Philosophy (PhD)	6	31.6
Doctor of Psychology (PsyD)	1	5.3
Master of Arts (MA)	4	21.1
Master of Science (MS)	4	21.1
Licensed Professional Counselor (LPC)	9	47.4
Licensed Clinical Alcohol & Drug Abuse Counselor (LCADC)	2	10.5
Other	6	31.6
Therapeutic modalities		
Individual	19	100
Group	7	36.8
Family	4	21.1
Couples	5	26.3
Peer support	1	5.3
Important parts of background or identity		
Ancestry or national origins	11	57.9
Gender	11	57.9
Race	11	57.9
Age	7	36.8
Religion	8	42.1
Where you live	5	26.3
Height	1	5.3
Weight	2	10.5
Another aspect of physical appearance	1	5.3
Sexual orientation	7	36.8
Education or income level	8	42.1
Physical disability	2	10.5
Shade of skin color	4	21.1
Tribe	3	15.8
Other	3	15.8
Ethnic-racial identity (*M* and *SD*)		
Assimilation	2.80	1.73
Miseducation	3.35	1.24
Self-hatred	3.22	1.65
Anti-dominant	2.53	1.86
Ethnocentricity	4.07	1.25
Multicultural inclusive	6.30	.96
Ethnic-racial salience	4.41	1.33

### Measures

#### Interview Discussion Guide

The authors developed a semi-structured discussion guide with prompts related to the TPB constructs. Participants were first asked about familiarity with cultural humility. Nearly all participants stated that they were familiar with the concept. Two participants were less sure about their familiarity, so they were provided a brief definition, emphasizing that it is a lifelong process that involves a mindset of being genuinely curious about someone else’s background or their cultural identity. Semi-structured questions were designed to elicit perceptions of cultural humility and perceived outcomes and expectations of integrating cultural humility into practice (behavioral beliefs). Participants were asked who would support or discourage their practice with cultural humility (normative beliefs) and about barriers and facilitators to implementing these practices into service delivery (control beliefs). Follow-up questions asked participants to rate their likelihood/motivation and confidence to practice with cultural humility in the future.

#### Quantitative Measures

Participants also completed a brief quantitative survey which assessed personal and professional demographics (e.g., age, gender, degrees obtained) prior to the interview. Additional quantitative measures were used to capture cultural identity and diversity among the sample, to help describe participants’ backgrounds and contextualize their views on cultural humility. Participants responded to a 15-item checklist (listed in Table 1), which asked them to indicate the most important parts of their identity. Finally, participants also completed the Cross Ethnic-Racial Identity Scale-Adult (CERIS-A).^23^ The CERIS-A is a 29-item measure of one’s attitudes related to their own racial and ethnic identity. For this study, Cronbach’s *ɑ* = 0.83. It includes seven subscales that reflect assimilation (emphasizing national identity over racial/ethnic identity), miseducation (stereotypes about racial/ethnic group), self-hatred (dislike of being a member of racial/ethnic group), anti-dominant (dislike of social majority group), ethnocentricity (how much racial/ethnic values inform day-to-day life), multiculturalist inclusive (strong connection to their own racial/ethnic group, with value for others’ groups and willingness to engage with them), and ethnic-racial salience (prominence of race in daily life). Each of the seven subscales contains four items, rated on a scale from 1 (strongly disagree) to 7 (strongly agree). Higher mean scores indicate greater endorsement.

### Data Analysis

Interviews were recorded and transcribed for analysis. The researchers used a thematic analysis approach to examine patterns and identify overarching themes within TPB constructs.^24,25^ The first author read through the interview responses and field notes (step 1, *familiarization*), then developed a code map based on TPB constructs. The code map consisted of primary coding categories for behavioral, normative, and control beliefs and ratings for motivation and confidence to practice with cultural humility (step 2, *generating initial codes*). There were also subcategories for each belief construct. For behavioral beliefs, this included general attitudes toward cultural humility and observed/expected outcomes from discussing culture with clients. Subcategories for normative beliefs included identified referents, both supportive and unsupportive, with ratings for willingness to engage in cultural humility with encouragement from others. Within control beliefs, subcategories for coding included barriers and facilitators to practicing with cultural humility. Two master’s level graduate research assistants (the second and third authors) independently coded transcribed responses. After the first round of coding, there was 78% agreement across interviews and Cohen’s kappa was 0.75, suggesting substantial agreement between coders. The first author reviewed code discrepancies, which were reconciled through discussion with the coding team. Only participant responses with coding consensus were used for analyses. After review, the first author read through categorized responses to identify patterns and underlying themes within each TPB construct (step 3, *searching for themes*). This was followed by *reviewing themes* (step 4) and incorporating additional relevant responses, reflecting subthemes across behavioral, normative, and control beliefs. All authors were involved in the process of *defining and naming themes* (step 5) and creating or reviewing the final report of results (step 6). Quantitative analyses were limited to descriptive statistics to help characterize qualitative responses and provide additional context for TPB constructs.

## Results

### Quantitative Results

Participants had been in practice for an average of 7.57 years (*SD* = 4.53, range = 2–17). Most participants (63.2%) saw clients part-time, and they predominately treated depression, bipolar, and mood disorders (100%), anxiety disorders (100%), and posttraumatic stress disorder (78.9%). Participants also reported on the diversity of their client sample (Supplemental Table 1). The categories of identity most highly endorsed were participants’ ancestry or national origins, gender, and race, followed by religion and education/income level. On the CERIS-A, the highest average score was observed for the *multiculturalist inclusive* subscale, indicating strong connection to one’s own racial/ethnic group, with value and respect for others’ groups (Table 1). This was followed by the *ethnic-racial salience* and *ethnocentricity* subscales, which suggest that race and ethnicity played a central role in participants’ personal lives.

### Behavioral Beliefs

Participants described positive attitudes and expectations about cultural humility, stating that it creates awareness and can lead to more healthy conversations. Overarching qualitative themes for positive behavioral beliefs included (1) improving case conceptualization, (2) enhancing the working alliance, and (3) breaking down barriers for clients from marginalized communities. However, they acknowledged some adverse outcomes that could potentially occur—although not outcomes that they actually had seen in practice. Themes for negative outcomes were (4) uncertainty in navigating practice and (5) negatively altering one’s relationship with the client in unintentional ways (Table 2).

**Table 2:** Qualitative themes within Theory of Planned Behavior (TPB) constructs for cultural humility

TPB construct	Themes	Subthemes
Behavioral beliefs	Improving case conceptualization	• Understand client health-related beliefs, and expectations
		• Process how cultural perspectives influence their current symptoms
	Enhance working alliance	• Improve rapport and working alliance
		• Avoid assumptions and biased first impressions
		• Allow for ongoing cultural discussions
		• Repair cultural ruptures when they occur
	Breaking down barriers	• Give space to discuss racism, discrimination, and social pressures
		• Help clients feel seen, heard, and safe
		• Be adaptive to client needs and preferences
	Uncertainty in navigating practice	• Knowing how to effectively engage clients
		• Identifying behavioral practices
		• Overpathologizing or fixating on a specific cultural attribute
		• Overcoming client biases
	Negatively altering relationship	• Client switches providers
		• Provider “others” client or makes them feel like a spokesperson
		• Conversations are off-putting or lead to ruptures
Normative beliefs	Agencies and clinical practices	• Provide exposure to multicultural frameworks, resources, encouragement
		• Less active support, standing productivity requirements, rote or boilerplate messaging
	Professional organizations	• Trainings, webinars, and professional development opportunities
		• Not an integral part of their missions
	University training programs	• Professors and instructors, coverage in ethics courses, students willing to learn and make changes
		• Completely optional
	Supervisors and mentors	• Supportive, with limitations
		• Unsupportive, low empathy, not willing to make treatment modifications
Control beliefs	Lack of support from systems	• No investment, providers are siloed, state level mandates on care, clinicians are stretched thin
	Personal limitations and strengths	• Lack of awareness, bias, negative attitudes are barriers
		• Personal willingness and contact with other groups are facilitators
	Professional development	• Limitations of current training materials and resources
		• Potential methods for improving trainings

#### Improving Case Conceptualization

Participants stated that adopting cultural humility into practice and asking clients about their cultural identities would help to “understand their worldview and their perspective better.” This led to better understanding of symptoms and presenting concerns. As Rachel described, “I find that I get a lot of information that helps me get to know the client better, but also, usually, it’s tied into the presenting problem in some way.” Arlo agreed, “I think outcomes are improved especially around if I can get a good sense of somebody’s health and illness beliefs and expectations, which are so culturally rooted, then I think I’m more able to support them.” Asking about values and beliefs helped to inform participants’ understanding of presenting concerns and the “things that they lean into for support… and for how they’re understanding what’s going on in their lives” (Mike).

Discussing clients’ cultural values and experiences also helped the clients to better process “aspects of themselves” and understand their symptoms. For example, Yvette described how discussing cultural identity can provide insight into “why certain experiences may be more hurtful or harmful than others,” and how these experiences relate to current symptoms. Finally, a broad exploration of culture may inform how clients’ shifting identities and “different aspects of who they are, at different points in times in their lives” can explain why some symptoms arise at unexpected times (e.g., when those identities become more salient). Exploring cultural identity also helped clients to process stigma and shame around mental health challenges, such as “an awareness of ‘not measuring up or not being faithful to’ perhaps something that was an expectation or a set bar within the family system” (Mike).

#### Enhancing the Working Alliance

Participants said that a cultural humility approach is “definitely a change from what we traditionally do in therapy,” but that it can make one’s practice more “powerful.” This was accomplished first by facilitation of trust, with rapport building being “supercharged” and “through the roof.” Second, participants said that cultural humility helped them to avoid biased impressions and assumptions about the client’s most salient identities, perceptions, and experiences—which was more likely when participants relied on a review of intake paperwork. Joe further described:

I just always think about just no set of cultures are a monolithic type of people. So even if you have somebody who looks like you, or they don’t look like you, you just always have to come from a place of curiosity and ask questions. Never just assume just because someone, how they identify ethnically or their gender… everybody is just different. And that’s not to say that there won’t be commonalities, but you have to come from such a place of curiosity and asking questions so you can be sure that you don’t say something racist, you don’t spout out a microaggression, you don’t offend the person.

Third, cultural humility allowed for ongoing discussions. Participants said that initially, providers may need to be flexible when clients are not willing to discuss their cultural identities, and “just gently put it out there” even if something seems “really obviously connected with their presenting issue.” However, asking these questions early on can continue to help, even if clients are initially hesitant. Participants said that, “they might not necessarily have much to say in the moment, but if it becomes part of what they’re bringing into session later, that they can feel comfortable talking about it, hopefully” (Rachel). In contrast, trying to discuss culture later on, when providers had not brought it before, was seen as a greater challenge. Finally, cultural exploration with clients could help participants to address cultural ruptures, including giving clients “agency in the relationship and that they can actually point out gaps if they see them” (Sarah). When mistakes happen, Joe recommended that providers “acknowledge and take accountability, responsibility for something like that because we’re human, we’re flawed. Things can happen.” Similarly, he described “Apologizing, owning up to it when you don’t know certain things, but also having a conversation around it too and bouncing back from that and going forward.” In contrast, participants said that some colleagues could become defensive, uncomfortable, or offended. When providers are unable to apologize and move forward, it creates “a huge disconnect. And then you find people leaving therapy, not going back to that same therapist, or feeling even more triggered than what they came in, or traumatized” (Joe).

#### Breaking Down Barriers for Clients from Marginalized Communities

Practicing with cultural humility, particularly talking about values, helped to improve connection and “permeate that wall of awkwardness when it comes to meeting with a stranger,” allowing clients from marginalized communities to feel safer and more seen and heard. When working with marginalized clients (e.g., “Black and brown people” and LGBTQ + people), cultural humility reportedly helped participants to validate their clients’ experiences of discrimination, give space for clients to express their anger about racism, and acknowledge “some of the social pressures that might be contributing to some of the problems that they’re having.” However, some clients may be “really resistant to talking about discrimination and barriers and oppression,” so providers should continue to be adaptive to client needs and preferences. For clients who did experience cultural stigma related to mental health, it was paramount for providers to operate with cultural humility: “If you’re already going in with a stigma or a preconceived notion and then you have a therapist who feed onto those stereotypes or doesn’t make you feel comfortable, or even more stressed, oh my God, that’s a disaster. That’s the disaster” (Joe).

#### Uncertainty in Navigating Practice

Despite the positive perceptions about cultural humility, participants expressed uncertainty about how to put these approaches into practice. They noted concerns about effectively engaging clients, such as ways to ask about culture or use appropriate terminology, with some risk of showing up in ways that “could make some clients feel unsafe in terms of disclosing their own identities.” Other potential limitations of practice were that providers may “struggle sometimes to clearly identify the behavioral anchors” which “dilutes the practice.” Finally, providers may overly focus on a single aspect of a person’s identity that is not salient, noting importance of “being careful as a clinician to not over-pathologize something in relation to culture and identity.” Ashley also stated:

But I think also a lot of times we miss the opportunity to explore what culture means to somebody, because we assume that if they look like us, or are the same religion, or same ethnic background or gender, that they have the same experiences that we do, or maybe think similarly to us—and that that is not always the case.

Some participants described experiences where it was difficult to engage with cultural humility because of elicited client biases (e.g., racism, misogyny). Sarah described working with clients “who had really explicitly sexist or misogynistic or machista views… That was something that I really struggled with in terms of maintaining a professional stance, because that was bringing up a lot of feelings for me.” Vanessa also described navigating a client’s biases, stating that “I didn’t want to just have that confrontation,” instead debating how to hold space, maintain positive regard, and gently reframe their biased thoughts. She further explained that in these situations, “You don’t have to agree with them. They’re still humans, and they still have feelings, and they are still worthy of [treatment]. Maybe if the situation is appropriate, having that dialog, you can find common ground.”

#### Negatively Altering Relationship with the Client

When integrating cultural identity and discussing cultural identity in therapy, participants identified as risk in unintentionally altering the client-provider relationship. There were some apprehensions about offending the client (e.g., by asking about cultural identity, saying something the wrong way). Participants were also concerned that naming differences (“cultural broaching”) would make clients want to work with someone from the same cultural background, if providers do not share the same experiences (e.g., not being a person in recovery, not having served in the military). Other risks of acknowledging differences were “othering the client” and making the client feel like a “spokesperson” for their racial, ethnic, or gender group. Rather, providers should signify that clients are not expected to teach them about their culture. Cultural conversations could also be “off-putting” to some clients who are not ready to explore it or if they have conflicting beliefs. Specifically, Alice said that for her practice in the South, “backlash” has not happened so far, but “there’s always a chance that it could, I think, especially around more traditional conservative mindsets of anti-intellectualism, anti-wokeness, things like that.”

### Normative Beliefs

Several participants said that “everyone” or “everybody” was supportive of practicing with cultural humility, and that they could not immediately think of anyone who would discourage cultural humility in practice. Yet, they acknowledged that there might be people who are not supportive, but “maybe not openly” or “behind closed doors” because “DE&I is too much of a buzzword… I think they’ll think it, but they won’t say it out loud” (Arlo). Brittany echoed this sentiment, that “it would be too controversial to say no.” Jessica also discussed how the larger social contexts have created pressures to avoid cultural humility work:

I live in the state of Florida, so we have a lot of current legislature that’s up to remove all mention of historical facts because it makes some people uncomfortable. And that, to me, is literally a spit in the face of cultural humility. … And so it’s very challenging to know how to navigate in a world where it’s likely not going to be a mandatory thing. And it might even be a thing that you could potentially be reprimanded for doing.

#### Agencies and Clinical Practices

Specific supportive referents included agencies and clinical practices. Agencies demonstrated support by providing exposure to multicultural frameworks, creating learning opportunities for cultural holidays, and giving space to consult about cultural issues. Mike explained how “over the past couple of years, there’s been a heavier push in terms of providing some resources on it” and encouragement. One agency was reported to hire clinicians that look like the population served and “making sure that [clients] match with a therapist that can understand them.” Additionally, some participants had colleagues or peer groups who provided support and encouragement for implementing cultural humility into their practice. This could be a “network of people that I kind of bounce off of” or people in their organization who provided indirect supervision and consultation.

In contrast, other participants said that their practices would be supportive, but provided fewer resources and less active guidance, “missing that extra step of initiating support.” Some agencies were perceived to focus on certification (e.g., training in manualized treatments) and regarding cultural humility, “there’s more they could do to be supportive of the practitioners at my private practice” (Ashley). Rose stated that:

I can’t think of anyone who would not be supportive of it. I just know that they certainly aren’t going to pay for me to be better trained in it. Particularly with the agency, where it would benefit them significantly if more people had training. So unless it was free, through the little online free thing that I had access to. If I really wanted to do a cultural humility training or learn more about it for their culture, they’d be happy if I went and did it, but… they wouldn’t pay for it. That’s not the kind of support that they’re willing to provide.

This participant also noted that her practice would not reduce productivity requirements to allow her to attend a training. When trainings were provided, participants reported no enforcement or follow-up to ensure that they were putting cultural humility into practice. Some participants expressed that “it was a buzzword, and we checked the box” but that trainings were rote. In another interview, Alice reinforced this idea, stating that at multiple agencies she worked with, trainings were “performative” and “they don’t really get into the nitty–gritty of any of it. They don’t have the difficult conversations. They’re not encouraging people to really look at their own biases.” At another agency, there was less diversity in the client population, “and so it’s not something that we talk about a lot either” (Rachel). Finally, Tracy compared her experiences at multiple clinical sites:

I think that at the previous clinic, I was able to really grow personally and professionally and develop my understanding of cultural humility and have really strong reflective conversations in supervision. I really value supervision, and I benefit from it, but my current experience with supervision is really negative. And it has felt like it’s just not a safe space. It feels like there’s very much a power dynamic and very didactic and doesn’t really allow me to think critically or to be reflective in the way that I wish I could.

#### Professional Organizations

Regional- and nationally-based professional organizations, such as the American Counseling Association and the Association for Multicultural and Diversity Counseling, were identified as supportive, offering trainings, webinars, and professional development opportunities. Professional organizations maintained a stance supporting cultural humility and multicultural practice, despite policies and legislation that discourage these practices. Still, participants said that for the most part, “this is something you have to seek out” and was not an integral part of organizational missions.

#### University Training Programs

Doctoral programs were identified as a supportive referent, particularly instructors and professors. Training programs discussed cultural humility and using a multicultural lens within ethics courses and provided additional resources for training. However, even though it was often a “purposeful activity,” it was still seen as “completely optional.” Some participants said that students were more willing to learn and make changes, whereas “when you’ve been more established, it can be a little harder to see yourself as part of the problem and recognizing some of the changes and sacrifices you might have to make” (Jalen). In contrast, learning these skills early on gave one participant more confidence to implement them as she established professional independence. This was seen as an individual responsibility (“more of it is going to have to fall on me to bring it up”), with less structured support for cultural humility as one progresses through doctoral and postdoctoral training. Arlo further explained:

You focus on the training of these cultural pieces early on just like any of the other benchmarks, but then once you get into the field, then there’s this unspoken, ‘You’re advanced enough in these areas of cultural and racial consideration, that you don’t need additional training on them.’ I think that comes out in subtle ways.

#### Supervisors and Mentors

Similar to agencies and training programs, clinical supervisors were perceived as supportive of cultural humility practice, but with limitations. For example, some participants said that they were unsure of whether previous supervisors would have been supportive, because they did not discuss cultural identity in any of their supervision sessions. Other supervisors did not introduce cultural discussions until George Floyd was murdered in 2020, or they waited until their trainees brought it up: “They’re willing to go there, to spend time with me on that, but they’re not taking the initiative” (Arlo). Still others described their supervisors’ attitudes as, “We’re not going to spend time during supervision talking about cultural humility or using the ADDRESSING framework, that’s something that you do on your own” (Brittany). Alice described a previous supervisor who actively discouraged cultural humility practice or disclosing elements of identity to clients:

He’s very low at empathy and just totally believes in the whole take-yourselves-up-by-your-bootstraps crap that we tell people. And he just didn’t really try to understand people’s culture, or he saw cultural factors of excuses almost like ‘they should just get over it’ kind of thing.

Some participants said their supervisors were not supportive, making “modifications in terms of understanding clients’ context and culture and environment. It’s very much like follow the module, just do exactly what it says. Don’t make any adjustments” (Tracy). She later expressed feeling that, “You just kind of have to keep your head down until you make your way up, until you’re the supervisor and you’re on the other side.”

#### Ratings of Motivation with the Support of Others

More than half of participants (*n* = 10, 62%) said that with the support of others from their agencies and clinical practices, professional organizations, training programs, supervisors, and mentors, they would be highly motivated to practice with cultural humility (i.e., rated their motivation as 10 out of 10). All other participants still rated themselves as 7 and above during interviews.

### Control Beliefs

Participants reported on barriers and facilitators to practicing with cultural humility. Identified themes were (1) lack of support from mental health systems, (2) personal limitations and strengths, and (3) professional development.

#### Lack of Support from Mental Health Systems

Participants stated that practicing with cultural humility was more difficult when their agencies and organizations did not invest in it, including fiscal support, making it a core value and part of the organization’s culture, and allotting time and effort to ensure that there is follow-through. Without this support, some participants felt “siloed in the work that we’re doing. And there haven’t been many opportunities to connect with other students or other interns or other professionals who are truly doing the work” (Tracy). Participants also explained that state-level mandates on service delivery created a barrier to effectively meeting clients’ needs, and the level of symptom severity and prioritizing client safety can “sometimes take on this level of urgency where it feels like things like culture and race become an afterthought or become less important” (Arlo). Finally, participants noted that “clinicians are stretched really thin and really busy,” which created a barrier for ongoing learning and practice.

#### Personal Limitations and Strengths

Participants’ lack of awareness, biases, attitudes (e.g., resistance to change), and rigidity of beliefs about how to provide services were personal barriers to practicing with cultural humility. Participants described noticing some providers having “monolithic views” and that “there’s just this one way to do it and one way to be.” Another participant described barriers when providers’ “perspectives are so closed off that they can’t see outside of themselves.” In contrast, participants endorsed that “having a willingness to do it” and contact with people from outside one’s own cultural group as facilitators for practicing with cultural humility.

#### Professional Development

Participants stated that most materials are “written for the majority” and do not encompass the diversity within practitioners. There was also the assumption that Black people, and providers from other marginalized communities, would feel comfortable talking about culture in training programs or supervision—although in truth, they were sometimes “uncomfortable” discussing these topics with non-Black colleagues and supervisors. Finally, trying to cover the breadth of diversity within and across cultural groups could be “overwhelming” or feel like “information overload.”

Several methods for improving resources were also identified, including handouts, videos, and other educational materials; trainings, webinars, and workshops from people with expertise about cultural experiences; and opportunities for continuous learning. Furthermore, participants described that ongoing consultation after trainings would be a facilitator, as well as being able to consult, discuss, and receive feedback from colleagues and receiving mentorship and “relational supports” that “holds me accountable.” Participants also expressed that cultural humility practice was easier when providers can find practical ways to integrate it into their existing clinical tasks and organizational trainings. Across the field, participants stated that shifting discourse has been a facilitator—namely, “it’s so much more towards the forefront than it was maybe even 10 years ago.” Furthermore, greater efforts should be made for providers who “refuse to be culturally inclusive,” and that “it doesn’t have to be confrontational. It doesn’t have to be belligerent or unprofessional, but we do got to call them out.”

### Ratings of Motivation and Confidence

During the interviews, five participants expressed very high motivation, rating themselves 10 out of 10 for starting or continuing to integrate cultural humility into their professional practice. However, a pattern emerged where several participants (*n* = 10, 62.5%) rated themselves high for motivation and intentions to practice but lower in confidence to do so. Across interviews, participants explained that their ratings for confidence were lower because they needed more resources and to increase knowledge and awareness. Lower confidence ratings were also due to lack of support, supervision, and check-in from their clinical organizations. Two participants stated that their confidence ratings were lower because they have room to grow. One participant rated herself as a 5 in both motivation and confidence, since cultural humility was competing with other tasks. Another rated herself nearly equal for both motivation and confidence in implementation (about 7 or 8) but reported slightly lower confidence in maintaining practices long-term (only 5 or 6). Only two participants rated themselves as highly confident, with a plan to implement but acknowledging room to grow.

## Implications for Behavioral Health

This qualitative study adds to the existing cultural humility literature by using the TPB to examine beliefs, norms, and facilitators and barriers to implementing cultural humility into practice. Findings reflected positive behavioral beliefs regarding cultural humility, including the potential for these approaches to improve case conceptualization, therapeutic alliance, and general practice, plus breaking down barriers for marginalized clients. Furthermore, most negative beliefs identified (i.e., uncertainty in navigating practice and altering one’s relationship with the client) were largely described as hypothetical, rather than direct past experiences. Together, these findings suggest that engaging in these approaches was viewed positively and perceived to be worthwhile. Furthermore, participants had high scores on the *multiculturalist inclusive* subscale of the CERIS-A, supporting the notion that this sample had a strong bond with their own cultural groups and respect for others’ cultural identities. The literature on cultural humility, and other multicultural approaches, has continued to grow, with multiple fields (e.g., social work, psychology, psychiatry, nursing) calling to increase training approaches.^26-28^ Yet, there are persistent gaps in training programs,^29^ and findings from this study elucidated why cultural humility may not be implemented as often as recommended.

### Professional and Organizational Supports for Cultural Humility Practice

Normative referents were agencies and clinical practices, professional associations, university training programs, and supervisors and mentors. While people and organizations were said to be actively supportive, participants also said that not many representatives would be willing to openly discourage cultural humility and multicultural practice. This was due, in part, to how strongly it has been recommended across health care fields, as well as the social importance placed on diversity, equity, and inclusion (DEI). Participants stated that the lack of organizational support may be more subtle (e.g., not allowing time or resources), rather than outright deterrents, such as policies prohibiting cultural humility practices. However, moving forward, there may be a greater likelihood of these prohibitions, given federal legislation restricting DEI-related activities and programs, particularly for university settings.

Limited allotment of training and resources may be due to the increasing mental health needs, which were rising prior to 2020 but were further exacerbated by the pandemic.^30,31^ At the same time, members of the behavioral health workforce have faced mounting burnout.^32^ These factors have contributed to a nationwide shortage in mental health providers.^33,34^ As it stands, it is not likely that mental health systems are currently positioned to be able to allocate resources for promoting cultural humility, including time, funding, and other structural supports. Yet, these approaches are sorely needed in order to resolve mental health inequities. Structural interventions are needed, including coordinated efforts to increase the number of available providers, retain providers in practice, and promote cultural humility across the field. These lessons should be reinforced through existing training structures.^4,35,36^ Notably, many accredited programs and professional organizations already have requirements for multicultural training. For instance, the American Psychological Association (APA) recommends incorporating diversity and multiculturalism throughout the course curriculums and clinical and research applications.^37^ Findings from this study suggest that there is a continued need for more support in this area, particularly for infusing cultural humility into all aspects of training. Mental health providers, including from trainees through leadership, must have access to resources and trainings that are practical, accessible, and effective, as cultural humility should not be relegated to an afterthought in service delivery.

### Individual-level Beliefs and Practices Regarding Cultural Humility

Similar barriers to cultural humility training and resources were echoed in control beliefs, where lack of support from mental health systems were compounded with personal limitations and shortcomings of existing training materials. Still, findings on beliefs and motivation reflected providers’ interest and willingness to engage clients with cultural humility, as well as desire for accountability and training in these approaches. In contrast, participants had lower ratings for confidence in their ability to practice cultural humility. Together, these findings would seem to indicate that well-designed, targeted trainings and resources to promote cultural humility would be well received by mental health providers, if allotted time and resources by organizations.

One critique of cultural humility is that its application can be vague.^38^ This can make it difficult to estimate how often, and to what degree, providers are adopting cultural humility. Creating a greater emphasis on behavioral outcomes of cultural humility (e.g., frequency of seeking feedback or consultation, integrating cultural assessments as part of standard intake procedures, tracking culture-related conversations via clinical documentation) may help to disambiguate whether, and how well, providers are engaging with cultural humility. For example, graded developmental approaches to using cultural formulation were used in one study to improve cultural humility among clinical psychology doctoral students.^38^ Even more importantly, providers should consider implementing client-related cultural humility measures throughout treatment, as we do with working alliance and other process variables. For example, the Cultural Humility Scale developed by Hook and colleagues^39^ is brief, accounts for multiple aspects of cultural humility, and has demonstrated good reliability and validity. Utilizing measures like these would allow for elicitation of client perspectives, with opportunities to address cultural ruptures—which can improve client outcomes.^12^ Providers can also assess their own perceived cultural humility, with measures such as the Multidimensional Cultural Humility Scale.^40^ While ratings of self-rated humility may be skewed,^38^ this may be a starting point to help providers compare with client perceptions and track their self-perceptions over time.

Despite its importance, there were some limitations to this study. First, providers who self-selected into the study may have been more comfortable talking about cultural humility. Second, given the nature of the topic, there is a high likelihood of social desirability bias in responding to both qualitative interview prompts and quantitative measures, although responses evidenced willingness to discuss their own shortcomings, concerns, and limitations. Third, while this study represents a diverse range of providers both personally (e.g., identities, regions of the country) and professionally, such as type of training and years in practice, this small sample size likely does not generalize to all providers. It is likely that there are additional beliefs and barriers that could be further explored. Furthermore, there are likely to be state-level differences, in part due to differing regional cultures and state-level licensure requirements regarding multicultural practice. Finally, interviewing mental health professionals in leadership roles may also expand understanding of the complexities in incorporating cultural humility into practice, which should be a goal of future research. Still, this study represents an important step to understanding mental health providers’ attitudes and intentions to practice with cultural humility, as well as underscoring the nuances of when and how these practices are used. Research has documented a direct relationship between cultural humility and working alliance.^41,42^ Findings from this study provide greater insight into the processes underlying that relationship and how it may be developed and maintained. It is vital to expand on training and implementation of cultural humility, as well as providers operating within a larger MCO framework, in order to improve provision of mental health services. This can help to reduce persistent and escalating mental health inequities, as well as improving treatment outcomes across a multitude of communities and cultural identity groups.

## Supplementary Information

Below is the link to the electronic supplementary material.

Supplementary file1 (DOCX 18 KB)

## Data Availability

Participants in this study did not give written consent for their data to be shared publicly, so due to the sensitive nature of the research, supporting data is not available.

## Declarations

### Reporting Guidelines

Consolidated criteria for reporting qualitative studies (COREQ).
