# Supplementary material for: Mental Health Providers’ Attitudes, Norms, and Beliefs About Cultural Humility in Service Delivery
Source: J Behav Health Serv Res. 2025 Jun 18;52(4):663–80. doi: 10.1007/s11414-025-09953-3 (PMC12528225; doi:10.1007/s11414-025-09953-3)
Supplement: Supplementary file 1 — Supplementary file1 (DOCX 18 KB) [file 11414_2025_9953_MOESM1_ESM.docx]

Supplemental Table 1

Clinician-Reported Percentage of Client Population by Group

|  | *M* | *SD* | Range |
| --- | --- | --- | --- |
| Race/ethnicity |  |  |  |
| Black and African American | 30.43 | 37.26 | 0-100 |
| Asian/Pacific Islander | 6.12 | 19.80 | 0-80 |
| Latino/Hispanic | 20.56 | 30.44 | 0-100 |
| American Indian/Alaska Native | .81 | 1.80 | 0-5 |
| White (non-Hispanic) | 37 | 38.82 | 0-100 |
| Biracial/multiracial | 4.75 | 6.85 | 0-20 |
| Other race or ethnicity | .31 | 1.25 | 0-5 |
| Disability status |  |  |  |
| Physical/orthopedic | 10.93 | 15.41 | 0-50 |
| Blind/visually impaired | .75 | 2.52 | 0-10 |
| Deaf/hard of hearing | .88 | 2.50 | 0-10 |
| Learning/cognitive disability | 5.87 | 8.77 | 0-20 |
| Developmental disability | 2.31 | 4.41 | 0-15 |
| Other type of disability | .94 | 2.02 | 0-5 |
| Gender identity |  |  |  |
| Cisgender female | 60 | 27.30 | 0-90 |
| Cisgender male | 32.37 | 26.76 | 0-100 |
| Transgender female | 1.25 | 2.89 | 0-10 |
| Transgender male | 2.25 | 3.60 | 0-10 |
| Non-binary/gender non-conforming | 3.43 | 7.68 | 0-30 |
| Other gender identity | .69 | 1.70 | 0-5 |
| Sexual orientation |  |  |  |
| Heterosexual | 70.81 | 30.54 | 13-100 |
| Gay or lesbian | 10.88 | 12.64 | 0-40 |
| Bisexual | 4.50 | 7.43 | 0-20 |
| Other sexual orientation | 2.31 | 5.43 | 0-20 |
| Unknown sexual orientation | 11.50 | 25.21 | 0-84 |
